# Improved Human Pluripotent Stem Cell Attachment and Spreading on Xeno-Free Laminin-521-Coated Microcarriers Results in Efficient Growth in Agitated Cultures

**DOI:** 10.1089/biores.2015.0010

**Published:** 2015-04-01

**Authors:** Alan Tin-Lun Lam, Jian Li, Allen Kuan-Liang Chen, William R. Birch, Shaul Reuveny, Steve Kah-Weng Oh

**Affiliations:** ^1^ Stem Cell Group, Bioprocessing Technology Institute, Agency for Science, Technology and Research (A*STAR), Singapore, Singapore.; ^2^ Institute of Materials Research and Engineering, Agency for Science, Technology and Research (A*STAR), Singapore, Singapore.

**Keywords:** attachment, laminin-111, laminin-521, microcarriers, pluripotent stem cells, spreading, xeno-free

## Abstract

Human pluripotent stem cells (hPSC) are self-renewing cells having the potential of differentiation into the three lineages of somatic cells and thus can be medically used in diverse cellular therapies. One of the requirements for achieving these clinical applications is development of completely defined xeno-free systems for large-scale cell expansion and differentiation. Previously, we demonstrated that microcarriers (MCs) coated with mouse laminin-111 (LN111) and positively charged poly-l-lysine (PLL) critically enable the formation and evolution of cells/MC aggregates with high cell yields obtained under agitated conditions. In this article, we further improved the MC system into a defined xeno-free MC one in which the MCs are coated with recombinant human laminin-521 (LN521) alone without additional positive charge. The high binding affinity of the LN521 to cell integrins enables efficient initial HES-3 cell attachment (87%) and spreading (85%), which leads to generation of cells/MC aggregates (400 μm in size) and high cell yields (2.4–3.5×10^6^ cells/mL) within 7 days in agitated plate and scalable spinner cultures. The universality of the system was demonstrated by propagation of an induced pluripotent cells line in this defined MC system. Long-term pluripotent (>90% expression Tra-1-60) cell expansion and maintenance of normal karyotype was demonstrated after 10 cell passages. Moreover, tri-lineage differentiation as well as directed differentiation into cardiomyocytes was achieved. The new LN521-based MC system offers a defined, xeno-free, GMP-compatible, and scalable bioprocessing platform for the production of hPSC with the quantity and quality compliant for clinical applications. Use of LN521 on MCs enabled a 34% savings in matrix and media costs over monolayer cultures to produce 10^8^ cells.

## Introduction

Human pluripotent stem cells (hPSC) such as human embryonic stem cells (hESC) and human-induced pluripotent stem cells (hiPSC) are expected to be used for cell-based therapeutics due to their ability for self-renewal and the potential to develop into various cell types.^1^ Clinical applications of these cells requires human doses of about 10^8^ to 10^10^ cells propagated in xeno-free defined conditions.^2^ To make such amounts of clinical-grade hPSC for therapy, there is a need to develop defined xeno-free large-scale production systems.

Our group focuses primarily on the development of long-term and robust scalable culture platforms for expansion of hPSC on microcarriers (MCs) for providing high cell yields in xeno-free defined conditions, while retaining pluripotency, normal karyotype, and tri-lineage differentiation ability.^3–7^ MC cultures can be easily scaled up in dynamic large-scale culture systems such as stirred bioreactors due to their 3D suspended nature. Moreover, high volumetric cell yields are achieved due to better oxygenation, better metabolite mass transport, as well as limited microenvironment toxicity.^5,8^ Recently, we described a defined, serum-free MC culture platform in which polystyrene (PS) MC are coated with cationic poly-l-lysine (PLL) polyelectrolyte and mouse laminin-111 (LN111). This platform supported stable propagation of hESC and hiPSC under continuous agitated conditions, achieving cell densities of 2.9±0.3×10^6^ cells/mL and a fold expansion of 14.3±0.4.^5^ These positively charged MCs with the mouse LN111 coating demonstrated a higher degree of cellular adhesion, spreading, and growth in agitated conditions than the VN-coated ones.^5^ Importantly, this platform produced a uniform size of cells/MC aggregates (∼300 μm) from single-cell seeding with high cell yields and enabled successful direct differentiation of these aggregates into cardiomyocytes (CMs).^5^ However, the mouse LN111 coating of the PS MC have several disadvantages; it leads to preaggregation of MCs before cell seeding, which affects their ability to form cells/MC aggregates and supports significant cell expansion in agitated conditions.^5,9^ An additional positive charge (PLL coating) is needed to support cell growth in agitated conditions^5^ and, most importantly, mouse LN111 is restricted for use in cell therapy since it is isolated from the mouse Engelbreth-Holm-Swarm sarcoma cells.^10^

Laminins (LNs) are a family of heterotrimeric glycoproteins comprising single α, β, and γ chains and are an integral part of all cell basement membranes. LNs are arranged in large (400–900 kDa) cruciform-shaped molecules with one long arm composed of segments of each of the three chains in a coiled-coil structure and two or three short arms each composed of the α, β, and γ chains.^11^ To date, five α, three β, and three γ LN chains have been identified to be associated with the forming of at least 16 LN isoforms. For instance, LN111 contains α1, β1, and γ1 while LN521 contains α5, β2, and γ1 (Table 1). These various LN isoforms are expressed in a tissue- and development-specific manner. LN-332 together with LN-521 or LN511 are present in epithelial basement membranes and lining surfaces of the body such as the skin, oral cavity, gastrointestinal and urinary tract, lungs, and different glands. LN-211 or -221 and LN-521 or -511 are present in muscle, nerve, and cardiac cells, and LN-411 or -421 combined with LN-521 or -511 are present in endothelial cells.^11–16^

**Table 1.** Comparison of LN521 Versus LN111-Coated Noncharged and Positively Charged MCs

*The coating LN*	*LN521*		*LN111*	
Chains	α5-β2-γ1		α1-β1-γ1	
Cell binding integrins	α6β1		α6β1	
	α3β1			
	Lutheran glycoprotein			
Binding affinity to integrin α6β1 (Kd)^9^	0.72±0.22 nM		9.5±3.3 nM	
Aggregation	No		Yes	

*Microcarriers*	*Noncharged MC^a^*	*Positive-charged MC^b^*	*Noncharged MC^a^*	*Positive-charged MC^b^*
2 h postseeding				
Cell attachment (%)	87.0±2.3	89.5±1.9	51.5±2.1	70.9±2.3
Cell attachment rate (×10^−2^/cm^2^/min)	1.53±0.06	1.75±0.16	0.99±0.05	1.20±0.05
5 h postseeding				
Cell spreading (%)^c^	84.8±2.3	87.3±0.9	14.5±2.1	58.6±7.3
After 7 days of growth in agitated plate cultures				
Cell density (×10^6^ cells/mL)	2.35±0.15	2.35±0.10	0.42±0.001	2.07±0.12
Tra-1-60 expression (%)	91.1±0.7	93.7±0.1	67.2±2.2	92.3±3.1
Cells/MC aggregates sizes (μm)	403±4	410±2	294±18	338±10
Increase in aggregate size per day (μm/day)	16±1	19±1	0.3±0.1	9±1
Concentration of aggregates (number/mL)	71±1	70±1	24±1	65±9
Increase in concentration of aggregates (number/mL/day)	4.0±0.1	4.0±0.1	1.0±0.1	3.0±0.4

^a^ Noncharged MCs: PS and Plastic MCs.

^b^ Positively charged MCs: PlasticPlus and PLL-coated PS and Plastic MCs (PLL+PS and PLL+Plastic).

^c^ The cumulative percentage of partial and fully spread cells.

MCs, microcarriers; LN, laminin; Kd, dissociation constant; PLL, poly-l-lysine; PS, polystyrene.

LN521 (α5β2γ1) and LN511 (α5β1γ1) are expressed early in the embryo development^11,17^ and were shown to support embryonic cells growth in monolayer plate (MNL) cultures.^15,18–20^ LN521 show a higher affinity to cell integrin α6β1 than LN111 (dissociation constant [Kd] 0.72±0.22 nM as compared with 9.5±3.3 nM [9]). Recent studies showed that LN521 (and not LN111) provides a robust substratum that enables efficient expansion of hPSC from single cells (without use of Rho-associated protein kinase [ROCK] inhibitor) in defined xeno-free medium.^18,19,21^ In combination with E-cadherin, LN521 also allows hPSC cell line derivation and development from blastocyst inner cell mass and a single blastomere cell without a need to destroy the parental embryo.^18,21^ LN521 also supports efficient transgene-free iPSC derivation and expansion, as well as directed differentiation to dopaminergic neurons under xeno-free conditions.^19^ More importantly, recombinant human LN521 can be produced in large amounts by an *in vitro* recombinant mammalian cell culture system as an abundantly available well-characterized human-origin protein.^20,22–24^ Due to the efficient performance of LN521 in supporting hPSC growth in MNL cultures and especially its high affinity to cell integrins, we postulate that it would also improve cell growth in agitated MC cultures and would enable growth on PS MCs without the additional need for positive charge. Moreover, by using human recombinant LN, we will be able to develop a xeno-free, GMP compatible system.

Thus, in this study, we compared hESC growth of LN111 and LN521-coated PS MCs in an agitated MC culture system. We demonstrated that LN521 (and not LN111) coating of PS MCs can support efficient hESC propagation in agitated cultures without the need for additional PLL positive charge coating. LN521-coated MCs support high efficiencies of cell attachment and spreading on MCs under agitation conditions, leading to regeneration of stable uniform-sized cells/MC aggregates and high cell yields. The expanded cells/MC aggregates were able to differentiate directly to the three germ layers as well as to beating CMs. In summary, we showed that the new xeno-free LN521-coated PS MCs culture platform is a simple, stable, and robust method for culturing hPSC under agitated conditions, amenable to scale up in controlled stirred bioreactors with compliance to Good Manufacturing Practice requirements.

## Materials and Methods

### Cell cultures, MCs, and matrices

hESC line HES-3 (ES Cell international) and induced pluripotent stem cell line IMR90 (generously provided by James Thomson [of ref.^25^]) were routinely maintained on Matrigel-coated tissue cultures in serum-free mTeSR™1 medium (StemCell Technologies), as previously described.^5^ Passaging (at a ratio of 1:10) of both cell lines was carried out by enzymatic dissociation of hESC colonies with dispase (StemCell technologies) (5 min at 37°C). The characteristics of the three MCs and three coatings used in this study are described in Supplementary Table S1. PS MC was purchased from Thermo-Fisher Scientific, and Plastic and PlasticPlus MCs were purchased from Solohill Engineering. Recombinant human LN521 (BioLamina), recombinant human LN111 (BioLamina), mouse LN111 (Life Technologies), and PLL (molecular weight of 70 kDa-150 kDa, PLL; Sigma-Aldrich) were used for MC coatings in these studies.

### Coating MCs with LN521, LN111, and PLL

Plastic and PlasticPlus MCs from Solohill Engineering were suspended in calcium- and magnesium-free phosphate buffer saline (PBS) and sterilized by autoclaving before use. PS MCs from Thermo-Fisher were also prepared in PBS but sterilized by gamma irradiation (10 min, 10 k Gray/h) as previously described.^5^ The different MC coatings were prepared by adding 20 μg of PLL, LN521, or LN111 to 22.5 mg of Plastic and PlasticPlus or 20 mg of PS MCs suspended in 1 mL PBS. In some conditions, a coating of PLL followed by LN521 or LN111 was prepared. The different types of coatings are fully described in Supplementary Tables S1 and S2. The coated MCs were washed with PBS twice, suspended in mTeSR1 medium (StemCell Technologies), and agitated at 4°C overnight before use.

### Protein surface density characterization

Surface density of LN111 and LN521 on the MC surface (noncharged or charged) was quantified by Ponceau S (Sigma) staining as described earlier.^4,5^ Briefly, 0, 10, 20, 30, 40, and 50 μg/mL of LN111 or LN521 in 600 μL PBS were incubated with 20 mg PS MCs (bare or coated with 1 mg/mL of PLL) in a 24-well plate at 4°C overnight. The adsorbed Ponceau S stain enabled a calculation of the ratio of protein adsorbed to the container versus that adsorbed to MC, for LN111 and LN521, respectively, at each concentration. This ratio was used to calculate the surface density of adsorbed LN111 or LN521, respectively, using depletion of protein from the depositing solution and based on an area of 11 cm^2^ for 20 mg of PS MCs.

### Measurement of cell attachment and spreading on the coated MCs

A single-cell suspension was prepared by dissociating confluent HES-3 from a 6-cm tissue culture dish with TrypLE™ Express (Invitrogen). One million viable cells from the single cell suspension were seeded into a single well of a six-well ultra-low attachment plate (Corning) containing 5 mL of mTeSR1 medium and 22.5 mg of coated Plastic and PlasticPlus or 20 mg of coated PS MCs. The plates were agitated on an orbital shaker at 110 rpm. Cell attachment was measured by quantification of viable unattached cells in aliquots of supernatant (NucleoCounter NC-3000; ChemoMetec), taken at the following times: 30, 60, and 120 min, after cell seeding. The percentage of cell attachment was calculated by subtracting the unattached cells from the initial viable cell concentration. Cell attachment rate was calculated using an exponential decay curve equation −ln(C_t_/C_0_)=*k*t,^26^ where C_t_ is the concentration of attached cells (cells/cm^2^) at time t, C_0_ is the original cell concentration (cells/cm^2^), and *k* is the rate constant (expressed in cells per cm^2^ per min).

**FIG. 1.**
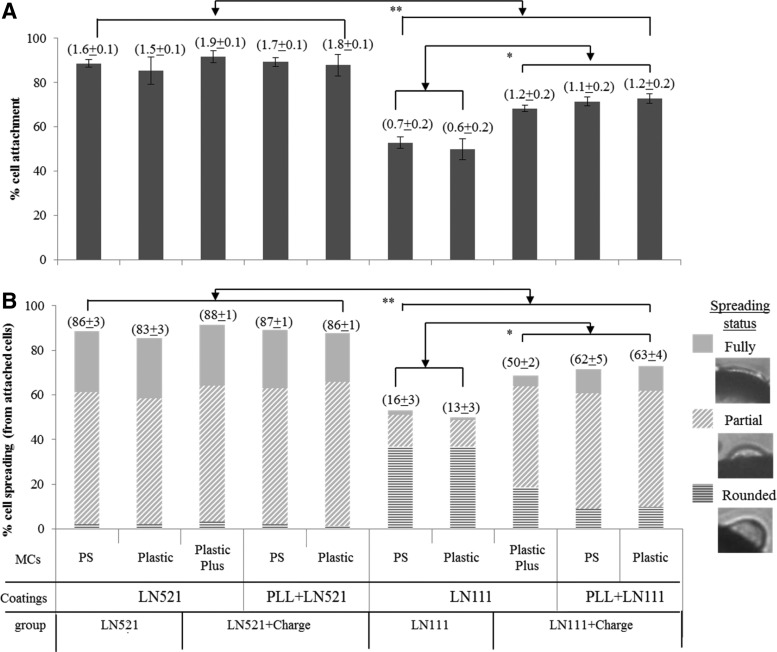
HES-3 cell attachment and spreading efficiencies on laminin-coated MCs (LN521, LN521+Charge, LN111, and LN111+Charge types). **(A)** Percentage of cell attachment, the rate of cell attachment (×10^−2^/cm^2^/min) is presented in brackets above the bars; **(B)** Percentage of rounded, partial, and fully spread cells (representative images in inset). The cumulative percentage of partial and fully spread cells is presented in brackets above the bars (%). * and ** indicate *p*<0.05 and *p*<0.01, respectively. LN, laminin; MCs, microcarriers; PLL, poly-l-lysine; PS, polystyrene.

Cell spreading was estimated by assessing the shape of the cells on the circumference of individual MCs (*n*>50) under phase-contrast microscopy. Cell spreading status was defined as no spreading (rounded), semi-spreading (partial), and flattened fully spread cell (fully) (Fig. 1).

### Cultivation of hESC on MCs in six-well agitated plates

A single-cell suspension of 1×10^6^ cells were seeded on 22.5 mg of coated Plastic or PlasticPlus or 20 mg of coated PS MCs suspended in 5 mL of mTeSR1 medium in six-well ultra-low attachment plates (Corning) (25 cells/bead). Cells were then cultured for 7 days under continuous agitation (orbital shaker, 110 rpm) at 37°C in a 5% CO_2_ incubator.

### Cultivation of hESC on MC in a spinner flask

Agitated MC cultures from six-well plates were seeded into a spinner flask as described in Lam et al.^5^ Briefly, the cells/Plastic MC aggregates obtained from continuous agitation plate cultures were mechanically dissociated into small cell clumps (about 250 μm). Ten million cells were seeded into a presiliconized (Sigmacote; Sigma-Aldrich) 100-mL spinner flask (Bellco) containing 25 mL of mTeSR1 medium and 225 mg of Plastic MC coated with LN521 (25 cells/bead). The culture was incubated at 37°C/5% CO_2_ in static condition for 24 h. The medium was then topped up to 50 mL, and the culture was agitated at 25 rpm. Eighty percent of spent medium was removed daily and replaced with fresh mTeSR1 medium.

### Cell counting

Viable cell density was measured by nuclei count method (NucleoCounter NC-3000; ChemoMetec) according to the manufacturer's instructions.

### Metabolites measurement

Glucose, glutamine, lactate, and ammonia concentrations in the cultures were measured using Bioprofile 100 plus (NOVA). Measurements were done by using both the spent media and the supplemented fresh medium after each media change. Specific consumption and production rates of the metabolites (q_m_) and yield ratios (Y_a/b_) were calculated as previously described.^27^

### Measurement of concentration and size hESC/MC aggregates

Twenty or more phase-contrast microscopy images of random areas (having at least 10 cells/MC aggregates) were taken from each MCs culture at days 1, 3, 5, and 7. The density of cells/MC aggregates was determined by counting the aggregates on each image (area of 2×2 mm) using the automatic cell counter plug-in of ImageJ 2.0 software (NIH).^5^ The change in the concentration of aggregates generated per day was calculated as follows: (N_day n_ − N_day n−1_)/(day_n−(day n−1)_), whereas N_day n−1_ and N_day n_ are the average aggregate concentrations at day n−1 and n, respectively. The size of the aggregates was estimated by calculating the square root of their area measured by NIH ImageJ 2.0 software.^5^ The change of aggregates size per day was calculated as (S_day n_ − S_day n−1_)/(day_n−(day n−1)_), whereas S_day n−1_ and S_day n_ are the average size of the aggregates at day n−1 and n, respectively.

### Cell entrapment

One (cells/MC) aggregate, obtained from 3 to 5 day-old LN-coated Plastic MC-agitated plate cultures (i.e., LN521, PLL+LN521, LN111, or PLL+LN111-coated Plastic MC), were transferred into a well of a 96-well ultra-low attachment plate. The plate was placed in an incubation chamber of Nikon Eclipse Ti microscope (Nikon Instruments, Inc.), where it was continuously video recorded by NIS Elements 4.10.00 software (Nikon Instruments, Inc.) over 24 h. Cell entrapment duration (from the point of contact of the free MC with the cells/MC aggregate, until it was completely covered by a layer of cells) was measured.^5^ The measurements were done in six replicates.

### Flow cytometry and immunochemical staining

The expression levels of extracellular surface marker Tra-1-60 in hESC populations were monitored by fluorescent flow cytometry as previously described.^4,5,7^ Briefly, a single-cell suspension was obtained by trypsin treatment and then filtered through a 40-μm sieve (BD Biosciences) to remove MCs. The cells were fixed with 4% paraformaldehyde (Sigma-Aldrich) and permeabilized with 0.1% Triton X (Sigma-Aldrich) in PBS. The cells were stained with 1:50 Tra-1-60 antibody (Millipore), and 1:500 dilution of goat anti-mouse, FITC-conjugated antibody (DAKO) was used as a secondary antibody. Measurements done using FACSVCalibur (Becton-Dickinson) were analyzed with FlowJo (Tree Star), and gating was selected at the point of intersection between the marker and its isotype control.

Immunochemical staining of Oct4 on cells/MC aggregates was carried out as previously described.^4^ Briefly, cells/MC aggregates were plated on LN521 coated plates and cultured for 2–3 days before fixing with 4% paraformaldehyde and permeablization by 0.1% Triton X in PBS. The cells were then stained with mouse primary Oct4 antibody (BD Biosciences) for 15 min. Alexa-fluor 594-conjugated F(ab′)_2_ fragment of goat anti-mouse IgG (Invitrogen) was used as a secondary antibody. A fluorescent mounting medium with DAPI (Vectashield) was added to cover the cells before imaging with a fluorescence microscope (Axiovert).

### Karyotype analysis

To assess chromosomal stability of the LN521-coated Plastic and PlasticPlus MC cultures after 10 passages, karyotyping of 20 colonies (using bromodeoxyuridine/colcemid) was performed by the Cytogenetics Laboratory at the Department of Obstetrics and Gynecology in Kandang Kerbau Women's and Children's Hospital, Singapore, as previously described.^4,7^

### Spontaneous *in vitro* differentiation

Spontaneous *in vitro* differentiation of HES-3 on LN521-coated Plastic and PlasticPlus MCs was carried out by embryoid bodies (EBs) formation, following the protocol described by Chin et al.^28^ Briefly, after 10 passages, cells/MC aggregates were cultured for 7 days in differentiation medium (KnockOut™DMEM [Life Technologies] with 15% fetal bovine serum [Life Technologies]) on a nonadherent dish. The cells were subsequently mechanically dissociated, re-plated on 0.1% gelatinized plates, and cultured for another 14 days at 37°C/5% in a CO_2_ incubator.

Immunochemical staining was carried out as previously described,^3–5^ to identify the differentiated cells from the three primary germ layers. Briefly, the re-plated differentiated cells/MC aggregates on the gelatinized plates were fixed (4% paraformaldehyde) and stained with primary antibody diluted in 1% bovine serum albumin/PBS; 1:400 for α-smooth muscle actin (SMA; Sigma-Aldrich), 1:250 for α-fetoprotein (AFP; Sigma-Aldrich), and 1:1000 for β-III tubulin (Millipore), for 1 h. Cells were then stained with FITC-conjugated anti-mouse antibody (Life Technologies) for another 2 h. After washing, a fluorescent mounting medium with DAPI (Vectashield) was added for 1 h before imaging with a fluorescence microscope (Axiovert 200M; Carl Zeiss).

Quantitative RT-PCR (qPCR) analysis was performed as previously published^3–5^ using primers specific for Oct4, AFP, GATA4, Hand1, Nkx2.5, PAX6, SOX1, and GAPDH. The qPCR reaction was carried on an ABI Prism 7500 Real-Time PCR (Applied Biosystem) using the following cycles: 50°C for 2 min, 95°C for 10 min, followed by 40 cycles of 95°C for 15 sec, and 60°C for 1 min. Data were expressed as a fold change of gene expression compared with that measured before HES-3 differentiation.

### Induction of CM differentiation

Direct CM differentiation was carried as previously described.^5,29,30^ Briefly, after cell expansion on Plastic MC, cells/MC aggregates were re-plated on LN521-coated plates. The cells were treated with 12 μM Gsk3 inhibitor CHIR99021 (Selleck) in RPMI/B27 without insulin (Life Technologies) for 24 h, followed by a treatment with 5 μM inhibitor of Wnt production-2 (IWP2; Stemgent) at day 3. The medium was replaced on day 5 by fresh RPMI/B27 without insulin and cells were then maintained in this medium until day 10, when they were maintained in RPMI/B27 with insulin (B27^®^ Supplement; Life Technologies) until day 15. The differentiated cells were then trypsinized into a single-cell suspension and fixed with 4% paraformaldehyde, followed by staining with 1:40 diluted anti-Cardiac myosin heavy chain (MF20; Developmental Studies Hybridoma Bank) and 1:200 anti-troponin 1 cardiac (cTnT; Millipore) before FACS analysis, as described earlier.

### Statistical analysis of data

All experiments were performed at least in triplicate. Data values are reported as a mean and standard deviation. Analysis of variance was used for a comparison between groups, with *p*<0.05 and *p*<0.01 considered as two levels of statistically significant differences.

## Results

Previously, we have shown that hPSC can grow in continuously agitated conditions on PS MCs coated with positive charge and mouse LN111.^5^ In this work, we have explored the possibility of using higher affinity recombinant human LN521 (Kd=0.72±0.22 nM, to integrin receptors^9,19^) as a sole coating of the MCs to develop a xeno-free, simpler, and scalable agitated culturing system. Adsorption isotherms of LN521 and LN111 coating on charged (PLL coated) and noncharged bare PS MCs are presented in Supplementary Figure S1. Similar trends of adsorption isotherms on the charged and noncharged MCs were observed for both LN521 and LN111. LN111-coated MCs showed protein surface saturation at higher levels than LN521. For the cell culture experiments, a protein concentration of 30 μg/mL of the coating solution was chosen. At this concentration, a saturation density of 550±3.2 and 676±0.7 ng/cm^2^ was achieved for the LN521 and LN111, respectively.

### Comparison of hESC cell attachment and spreading on LN111- and LN521-coated MCs

Several polystyrene MCs (PS, Plastic, and PlasticPlus) were used to explore the effect of LN521 or LN111 coatings as well as the effect of additional positive charges on hESC growth in agitated conditions (Supplementary Tables S1 and S2). Positive charge was introduced onto the MCs by either coating of noncharged MCs (PS and Plastic) with PLL or using positively charged commercial MCs (PlasticPlus). The different MCs were divided into four groups: (1) LN521 : LN521 coated on PS and Plastic MCs without positive charge; (2) LN521+Charge: LN521 coated on positively charged MCs. These are PLL+PS, PLL+Plastic, and PlasticPlus MCs; (3) LN111: LN111 coated on PS and Plastic MCs without positive charge; and (4) LN111+Charge: LN111 coated on positively charged MCs. These are PLL+PS, PLL+ Plastic, and PlasticPlus MCs (Supplementary Tables S1 and S2).

LN111 MCs (LN111-PS and LN111-Plastic) coated with native mouse LN111 as well as recombinant human LN111 aggregated substantially in cell culture medium before cell seeding (Supplementary Table S1). This phenomenon that was previously observed^5,31^ can be attributed to aggregation of the LN111 due to binding interactions between its three short arm structure.^31,32^ Preaggregation of MCs before cell seeding resulted in low cell attachment and spreading efficiencies (only 51.5%±2.1% of the cells attached and 14.5%±2.1% spread, Table 1). This preaggregation phenomenon can be prevented by addition of PLL coating or by using positively charged MCs (PlasticPlus) (Supplementary Table S1). LN111+Charge MCs do not preaggregate probably since the LN molecules are tightly bound to the positive charges on the MC surface, thus restricting their freedom to self-assemble. These MCs (LN111+Charge) achieved higher cell attachment (70.9%±2.3%) and spreading (58.6%±7.3%) efficiency than the LN111 ones (*p*<0.5, Fig. 1A, B), probably due to the additional effect of electrostatic attraction of the negatively charged cells to the positively charged MCs.^5^

Interestingly, a coating of LN521 on noncharged or positively charged MCs did not result in preaggregation of the MCs (Supplementary Table S1) probably due to lower strength of the intra-molecular interactions required for polymerization of LNs.^33^ Moreover, in contrast to the LN111-coated MCs, cell attachment and spreading on LN521-coated MCs was not affected by the positive charge of the MCs. Similar high cell attachment efficiencies (87.0%±2.3% and 89.5%±1.9%, Table 1 and Fig. 1A) and attachment rates (1.53±0.06×10^−2^ and 1.75±0.16×10^−2^ cells/cm^2^/min, Table 1 and Fig. 1A) as well as high spreading efficiencies (84.8%±2.3% and 87.3%±0.9%, Table 1 and Fig. 1B) were observed using both positively charged (LN521+Charge) and noncharged LN521-coated MCs. The extent of cell attachment and spreading on the LN521-coated MCs were significantly higher than the values measured with the LN111-coated ones (*p*<0.01, Fig. 1A, B). The hESC attach at a faster rate to LN521-coated MCs, probably due to the higher affinity to cell integrin^9^ and thus additional positive charge was unnecessary.

In summary, LN521 coating of MCs efficiently supported hESC attachment and spreading in agitated conditions, without the need for additional positive charges.

### Cell growth and kinetics of cells/MC aggregates evolution on LN111- and LN521-coated MCs in agitated cultures

**FIG. 2.**
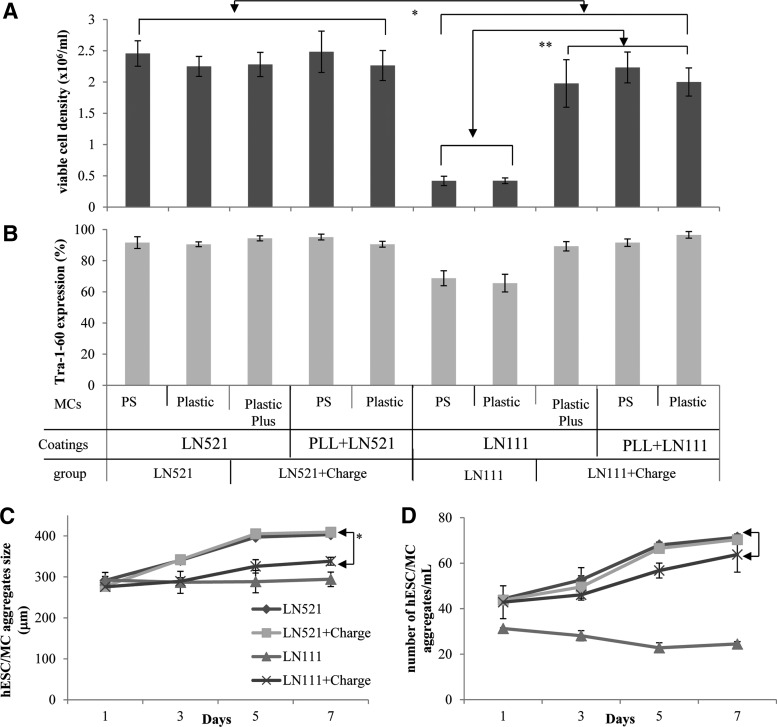
HES-3 growth on LN-coated MCs (LN521, LN521+Charge, LN111, and LN111+Charge types) in agitated plate cultures. **(A)** Cell density after 7 day of propagation. **(B)** Percentage of cells expressing Tra-1-60 after 7 days of growth. **(C, D)** Evolution of hESC/MC aggregate size and concentration during cell growth. * and ** indicate *p*<0.05 and *p*<0.01, respectively. hESC, human embryonic stem cell.

One of the critical factors in achieving high cell yields in agitated hESC cultures is the initial formation of cells/MC aggregates and their evolution during cell propagation.^5^ Thus, we have followed kinetics of aggregate formation (concentration and size) in LN521- and LN111-coated MC cultures and tested their effect on cell growth (Table 1 and Fig. 2C, D).

The noncharged LN111-coated carriers (LN111) that preaggregate and support low cell attachment and spreading did not form significant amounts of cells/MC aggregates. Aggregate concentration (24±1 aggregates/mL) and size (294±18 μm) were maintained relatively constant throughout the cell cultivation, showing a nonsignificant increase in concentration (1.0±0.1 aggregates/mL/day) and size (0.3±0.1 μm/day). The lack of ability to generate significant amounts and large enough cells/MC aggregates (above 300 μm in size^5^) during the first 1–2 days of growth resulted in low cell yield (0.42±0.001×10^6^ cells/mL, only two-fold expansion) and pluripotency (67.2%±2.2%) after 7 days of cultivation (Fig. 2A, B). Addition of positive charges to the LN111 carriers (LN111+Charge) that improved cell attachment and spreading performance resulted in the gradual buildup of cells/MC aggregates concentration (3.0±0.4 aggregates/mL/day) and size of (9±1 μm/day), leading to the generation of a high concentration (65±9 aggregates/mL) of large-sized (338±10 μm) aggregates at day 7 of growth. The improved ability to generate aggregates resulted in high cell yields (2.07±0.12×10^6^ cells/mL, 10.5-fold cell expansion) and pluripotency (92.3%±3.2%) (Fig. 2A, B).

All LN521-coated MCs (noncharged and positively charged) had high attachment and spreading efficiency (Fig. 2A, B). A gradual increase in aggregate concentration (4.0±0.1 aggregates/mL/day) and size (18±2 μm/day) resulted in 71±1 aggregates/mL with cell/MC aggregates of 406±4 μm at day 7 of growth (Table 1). The aggregate buildup in the LN521-coated carriers was more efficient than the LN111+Charge MCs in size (∼20%, *p*<0.05, Fig. 2C) but not in concentration (∼10%, Fig. 2D). Subsequently, a 13% increase in cell yield (*p*<0.05), 2.35 versus 2.07×10^6^ cells/mL (Table 1), was observed.

**FIG. 3.**
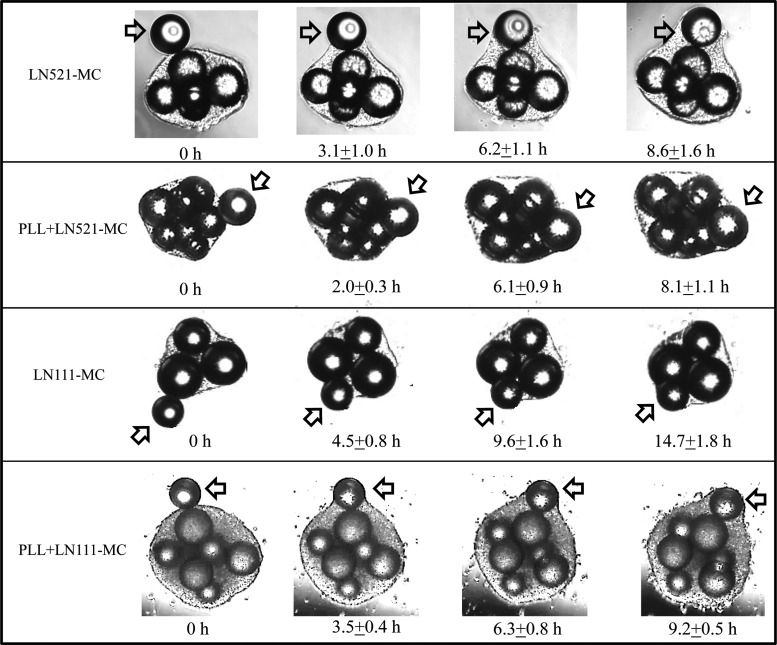
Time-lapse images of LNs (LN521-, PLL+LN521-, LN111-, and PLL+LN111-)-coated single Plastic MCs being incorporated into cells/MC aggregates, the arrow indicating the particular MC being incorporated.

To further explore the efficiency of cells/MC aggregates built up in LN521- or LN111-coated MCs systems, time-lapsed images of a single extracellular matrix (ECM)-coated MC (LN521, LN111, PLL+LN521, and PLL+LN111) being incorporated into a pre-existing cell/MC aggregate was followed (Fig. 3). All single ECM-coated MCs were incorporated into the cells/LN521-Plastic aggregate within 15 h (time from the point that the MC touched the cells/MC aggregate until it was fully engulfed by migrating HES-3). The LN521- and PLL+LN521-coated MCs were engulfed by the cell/MC aggregate within 8.4±0.4 h. In comparison, LN111-coated MCs were engulfed at a slower rate, which was dependent on the additional positive charges (PLL+LN111: 9.2±0.5 h vs. LN111: 14.7±1.8 h).

The earlier results show that LN521 is advantageous compared with LN111 in supporting hESC cell growth in MC-agitated cultures. It supported higher cell attachment, spreading rates, and cell yields. Furthermore, the rate of MC engulfment was significantly higher, and no MC preaggregation was observed. Thus, we further investigated long-term cultures and differentiation capability of hESC using LN521-coated MCs.

### Long-term expansion of hESC on LN521-coated MCs

**FIG. 4.**
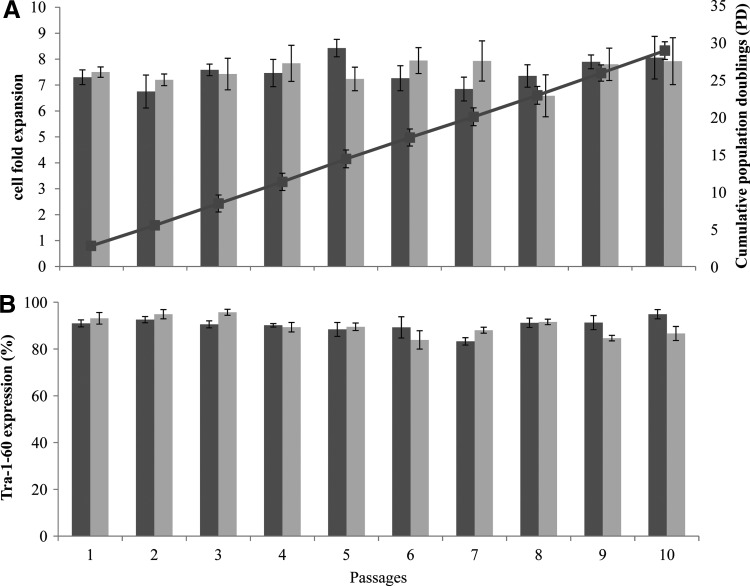
Long-term MC cultures of HES-3 propagated in mTeSR™1, on LN521-Plastic (dark gray) and LN-521-PlasticPlus (light gray) MCs in static condition. **(A)** Cell fold expansion and the cumulative population doublings and **(B)** percentage of Tra-1-60 expression during 10 successive weekly passages.

**FIG. 5.**
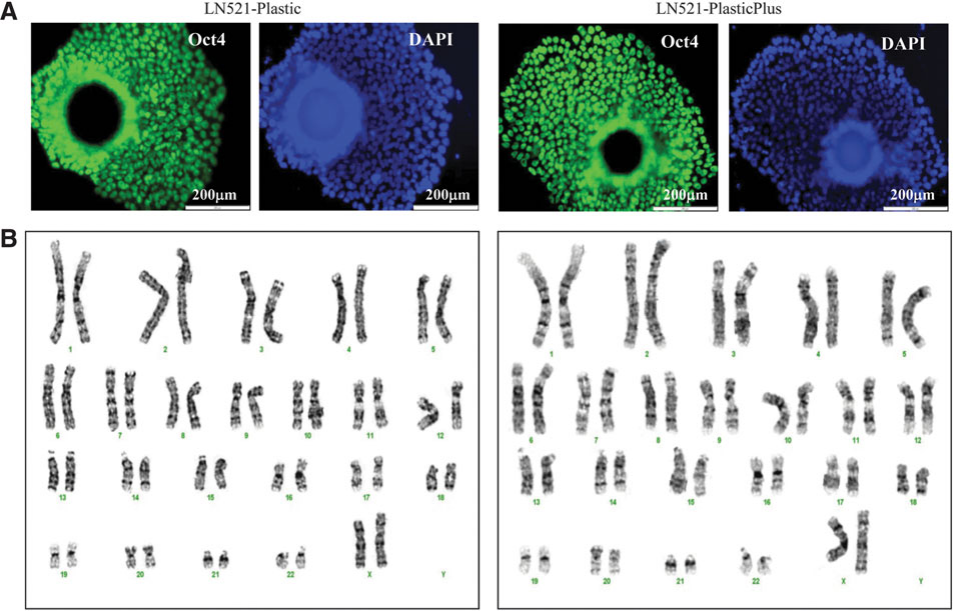
Characterization of HES-3 expanded on LN521-Plastic and LN521-PlasticPlus MCs after passage 11 of the culture. **(A)** Oct4 immunochemical staining of cells/MC aggregate and **(B)** normal 46 XX karyotypes by G-banding, 20 metaphase spreads were counted per sample.

To evaluate the robustness of hESC growth on LN521 and LN521+Charge MCs, we have propagated HES-3 cells in static cultures for 10 sequential passages on both types of MCs, LN521-coated Plastic MCs and LN521-coated PlasticPlus MCs in defined serum-free mTeSR1 medium. We have found that HES-3 can be cultured on both MCs for at least 10 passages. Similar high cell expansion (∼7.5-fold, Fig. 4A) and expression of Tra-1-60 pluripotent marker (>90%, Fig. 4B) were maintained throughout the 10 passages. The cumulative population doublings of cells at passage 10 was 29 (Fig. 4A). At passage 11, the culture was confirmed to have a normal karyotype (Fig. 5B) and immunochemical staining confirmed high expression levels of Oct4 protein (Fig. 5A).

In summary, both LN521-coated Plastic and LN521-coated PlasticPlus MCs were shown to support long-term HES-3 self-renewal capability in defined serum-free conditions. Since positive-charged MCs can result in nonspecific adsorption of different negatively charged proteins and dyes used in a variety of assays, we have chosen the noncharged Plastic MC for further exploration.

### Scaling up of HES-3 expansion on LN521-coated noncharged (LN521-Plastic) MCs in stirred spinner flask cultures

To demonstrate the scalability of LN521-Plastic MC cultures, HES-3 cultures were scaled up from a 5-mL suspended MC-agitated plate culture surface followed by a 50-mL stirred spinner using a split ratio of 1:10.

**FIG. 6.**
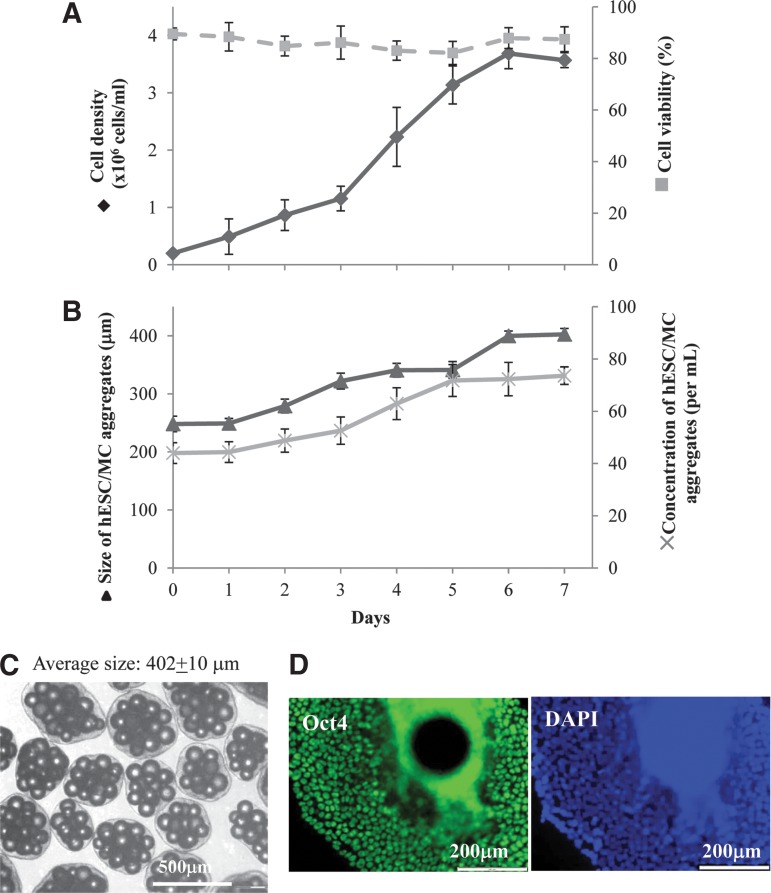
Scaling up of LN521-Plastic HES-3 MC cultures in a stirred spinner flask. **(A)** Kinetics of cell growth, cell density (♦), and viability (▪). **(B)** Evolution of cell/MC generation during growth, size (▴), and density (x). **(C)** Representative figure of cells/MC aggregate and average aggregate size at day 7. **(D)** Oct4 immunochemical staining of cells after 7 days of propagation.

A 3 day lag period (Fig. 6A) was needed for the establishment of a critical initial size of cell/MC aggregates (>300 μm, Fig. 6B) that were previously shown to be essential for supporting further cell expansion.^5^ Exponential cell growth started between day 3 and 6, with a doubling time of 26±2 h (Supplementary Table S3), resulting in 74±3 cell/MC aggregates/mL with sizes about 402±10 μm (Fig. 6B, 6C and Supplementary Table S3) and a cell yield of 3.56±0.13×10^6^ cells/mL with a viability of 90% (Fig. 6A and Supplementary Table S3). High levels of pluripotency were demonstrated by flow cytometry (>90% of Tra-1-60 expressing cells, Supplementary Table S3) and Oct4-positive immunostaining (Fig. 6D).

Specific metabolite consumption rates (Supplementary Table S3) of glucose (qGlc) and glutamine (qGln) were 0.40±0.01 and 0.038±0.003 mmol/10^9^ cells/h, respectively; whereas the specific waste product production rates of lactate (qLac) and ammonia (qAmm) were 0.79±0.03 and 0.031±0.001 mmol/10^9^ cells/h, respectively. Thus, the yield ratio of the metabolites of lactate/glucose (Y_Lac_/_Glc_) was 1.91 and that of ammonia/glutamine (Y_Amm_/_Gln_) was 0.83, which are in the expected range for hESC under normal glycolytic metabolism.^27^

**FIG. 7.**
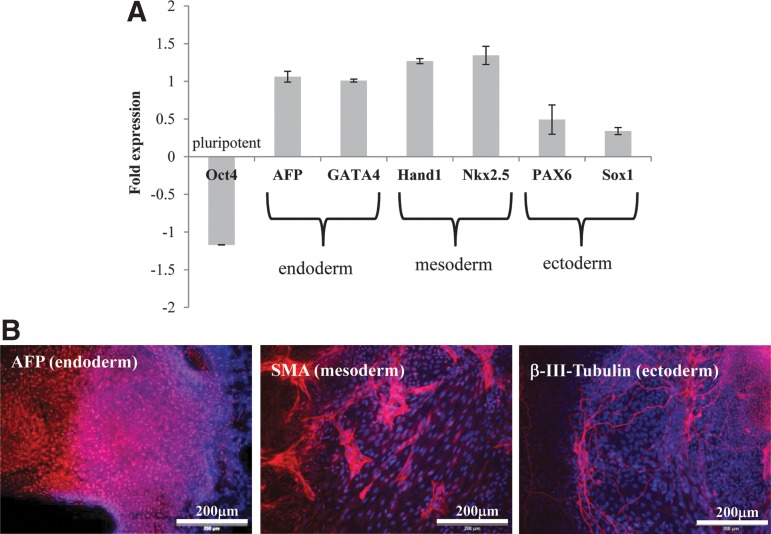
Characterization of spontaneous embryoid body-based differentiation of HES-3 cells expanded in spinner flasks on LN521-Plastic MCs. **(A)** Fold change of pluripotent and three germ-layer-specific genes compared with undifferentiated HES-3 (quantitative RT-PCR). **(B)** Representative images showing cells expressing AFP (endoderm), SMA (mesoderm), and β-III tubulin (ectoderm). Scale bar indicates 200 μm. AFP, α-fetoprotein; SMA, α-smooth muscle actin.

Real-time PCR analysis (Fig. 7A) and immunochemical staining of *in vitro* EB-differentiated cultures (Fig. 7B) demonstrate the tri-lineage differentiation capability of the HES-3 cells cultured on LN521-coated Plastic MCs. Moreover, cells/MC aggregates were directly differentiated to CMs using the Wnt-modulation protocol. After 10–12 days of differentiation, contractile aggregates were observed and 53.2%±2.9% and 59.5%±1.1% of the final cell population was positive for MF20 and cTnT, respectively (Supplementary Fig. S2).

### Growth of hiPSC on LN521-coated MCs

To demonstrate the universality of the LN521-coated MCs system, an additional hiPSC cell line (IMR90) was propagated on LN521-Plastic and LN521-PlasticPlus MCs in agitated plate cultures. About 1.85×10^6^ cells/mL (approximately nine-fold expansion) of pluripotent (>90% of Tra-1-60) cells were achieved within 7 days of growth on LN521-coated MCs (noncharged or positively charged) (Supplementary Fig. S3).

In summary, we have developed a robust, xeno-free, scalable, and GMP-compatible MC culturing system. PS MCs coated with recombinant human LN521 (without additional positive charges) can support efficient cell attachment, spreading, and growth in agitated conditions, and maintain pluripotency for more than 10 passages. The cultured cells retained normal karyotype and the ability to differentiate into lineages of all three germ layers and specifically to CMs.

## Discussion

A defined xeno-free scalable system for hPSC expansion and differentiation is necessary for hPSC application in clinical cell therapy. Toward that end, progress has been made in developing defined xeno-free substrates for long-term hPSC self-renewal in defined media in conventional 2D MNL cultures.^2,18,21,34–36^ This article further explored the use of these xeno-free substrates for the development of a robust scalable MC cultures for large-scale production of hPSC. Previously, we reported that coating of PS MC with native mouse LN111 and cationic PLL maintained the growth and pluripotency of hPSC in serum-free medium in MC-agitated cultures. However, this system had several disadvantageous: (1) self-aggregation of LN111-coated MCs before cell seeding; (2) requirement of additional cationic coating to support cell attachment, spreading, and growth; and (3) the mouse-origin substrate, which limits its use for clinical applications. By using xeno-free recombinant human LN521, we have overcome these disadvantages. Xeno-free LN521-coated MCs, which do not preaggregate, supports efficient hPSC cell attachment, spreading, and expansion under agitated MC cultures with defined serum-free medium. These cultured pluripotent stem cells were able to form derivatives of the three germ layers *in vitro* and also able to directly differentiate into CMs under serum-free conditions. Pluripotent hPSC were maintained on LN521-coated MCs for at least 10 passages under defined culture conditions and still maintained high expression levels of stem cell markers. Taken together, these results demonstrate that LN521-coated MCs are a better support for xeno-free, serum-free hPSC in large-scale, long-term MC cultures than LN111.

Native mouse and recombinant human LN111 coated on PS and Plastic MCs (noncharged) result in MC aggregation; whereas LN521-coated MCs do not aggregate. Self-aggregation (or self-assemble) of LNs into large polymers is one of the key steps for the formation of basement membranes.^33,37^ Previous studies have shown that LN111 self-assembles into a polymer by the interaction of the three short arms (α1-, β1-, γ1-) *via* the LN-domain VI, also known as the three-arm interaction model,^32,37,38^ where there are strong interactions between α1-α1, α1-β1, β1-β1, and β1-γ1,^31,33,39,40^ whereas self-assembly might not occur in LN521 since it does not compose of α1- and β1- arms. This tendency of LN111 to self-aggregate presumably occurred during the MCs coating in serum-free medium that contains 50 μM Ca^2+^ and at 21–35°C, with LN111 concentration >60 nM.^41,42^ These conditions favor the polymerization process. Addition of positive charges on the MC surface is assumed to lead to the tight binding between LN-domains and the polyelectrolytes, thus limiting the freedom of the LN-domains to extend from the surface and preventing MC preaggregation.^5^

LN521, on the other hand, which is composed of α5, β2, and γ1 (Table 1), has a lower tendency to aggregate.^43^ Although reports have shown that α5-α5 self-interact in a manner similar to that of the α1 domain,^33^ α5-β2 cannot engage in intermolecular interactions and α5 cannot bind to γ1, leading the self-assembly only by the transient contacts between the β- and γ-short arms and consolidated by the slower incorporation of a α-short arm.^43^ Therefore, no self-aggregation of LN521-coated MC was observed. The prevention of MC preaggregation is extremely important, since it impacts the ability to form cells/MC aggregates and support expansion of the cells.^5^

The primary factor for cell growth on MC is the initial cell attachment and spreading, where single hESC should adhere well to the MC surface within 1–2 h, and spread within 5–6 h to form a homogeneous distribution around the MC.^5,44^ Otherwise, hESC will tend to aggregate as EBs and undergo spontaneous differentiation.^45,46^ Cell attachment to the MC surface under agitation conditions, which needs a high-affinity interaction between the cells and MCs, can be achieved either by using an ECM protein coating that interacts with the cell integrins^47^ or by positive charges that involve electrostatic interactions with the negatively charged surfaces of the cells,^48^ or both.^49,50^ The LN111 coating of MCs requires an additional positive charge to ensure cell attachment spreading and growth in agitated conditions,^5^ while LN521 as a single coating can achieve significantly higher cell attachment spreading and growth than the PLL+LN111 MCs (Fig. 1A, B). LNs interact with cells and control cell growth and differentiation through their binding to integrins, mainly isoform α6β1.^11^ Reports showed that LN521 has a high binding affinity for integrin isoform α6β1 (Kd=0.72±0.22), while LN111 only has modest affinity for α6β1 (Kd=9.5±3.3)^9^ (Table 1). Moreover, unlike α1 chain, the α5 chain can interact with other LN receptors such as the Lutheran glycoprotein, which is known as basal cell adhesion molecule.^51^ Besides, the β2 chain also has a higher binding affinity for integrin α3β1 than the β1 chain.^52^ Collectively, this accumulating evidence confirmed that LN521 promote more efficient cell attachment, spreading, and growth compared with LN111. Notably, changes in type (noncharged vs. positively charged), porosity (porous vs. nonporous), and size (97 vs. 120 μm) of MCs (Supplementary Table S1) did not affect cell attachment and spreading as well as the final cell yields (Table 1).

Growth kinetic and aggregation evolution between cells on LN521-Plastic (current results) and PLL+LN111-PS MCs (previous results^5,30^) in spinner flask cultures were compared (Supplementary Table S3). Overall, LN521-coated MCs showed improved kinetics of cell growth compared with PLL+LN111-coated MCs. The LN521-coated MCs spinner culture demonstrated shorter lag period (2 days, Fig. 6A, vs. 4 days^5^) and shorter duration of cultivation (6 days, Fig. 6A vs. 8 days^5^). Similar to the results obtained in agitated plate culture (Table 1), higher concentrations of cell/MC aggregates were generated (74±3 vs. 54±3) and larger sizes aggregates (410±4 vs. 316±11 μm) were obtained in LN521-coated MCs after 7 days of growth. Higher, although not significant, final cell yield (3.6±0.1 vs. 3.1±0.4×10^6^ cells/mL) was harvested at day 7. Similar doubling time (25.8±1.8 vs. 26.6±3.2), specific metabolite consumption and production rates, and expression of Tra-1-60 pluripotent marker (>95%) were obtained in both MC cultures (Supplementary Table S3).

In comparison to the nonhomogenous conventional monolayer cell culture, MC cultures have a suspended homogenous nature and thus can be scaled up in a stirred bioreactor and controlled on line for environmental parameters (e.g., dissolved oxygen, pH).^34^ Moreover, a lesser amount of LN521 (Supplementary Table S4) is needed to generate 10^8^ cells for cell therapy.^2^ Therefore, it is possible to reduce the cost of LN521 and still maintain high yields with MC cultures. Besides, due to the lower amount of medium required to feed the cells over the entire culturing period compared with monolayer cultures, this leads to a total saving of 34% in MC compared with monolayer cultures. Further saving in medium cost can be achieved by optimization of medium feed (e.g., fed-batch or perfusion). In this work, cell harvesting from the MCs was done by enzymatic dissociation followed by a 40 μm sieve to remove the MCs. For a larger scale, a special system should be developed. In conclusion, our results illustrated that a defined xeno-free LN521 matrix coated on noncharged or positively charged MCs can support efficient hPSC attachment, spreading, and expansion, as well as directed differentiation under serum-free conditions. Our xeno-free and serum-free MCs culture system could allow cost-effective, GMP-compliant production of large amounts of clinical-grade hPSC with consistent and predictable characteristics to achieve the needs in cellular therapy.

## Supplementary Material

Supplemental data

Supplemental data

Supplemental data

Supplemental data

Supplemental data

Supplemental data

Supplemental data

## Abbreviations Used

AFP: α-fetoprotein
EBs: embryoid bodies
hESC: human embryonic stem cells
hiPSC: human-induced pluripotent stem cells
hPSC: human pluripotent stem cells
Kd: dissociation constant
LNs: laminins
MCs: microcarriers
PBS: phosphate buffer saline
PLL: poly-l-lysine
PS: polystyrene
qPCR: quantitative RT-PCR
SMA: α-smooth muscle actin
