# Supplementary figures and images for: Improved Human Pluripotent Stem Cell Attachment and Spreading on Xeno-Free Laminin-521-Coated Microcarriers Results in Efficient Growth in Agitated Cultures
Source: Biores Open Access. 2015 Apr 1;4(1):242–57. doi: 10.1089/biores.2015.0010 (PMC4540119; doi:10.1089/biores.2015.0010)

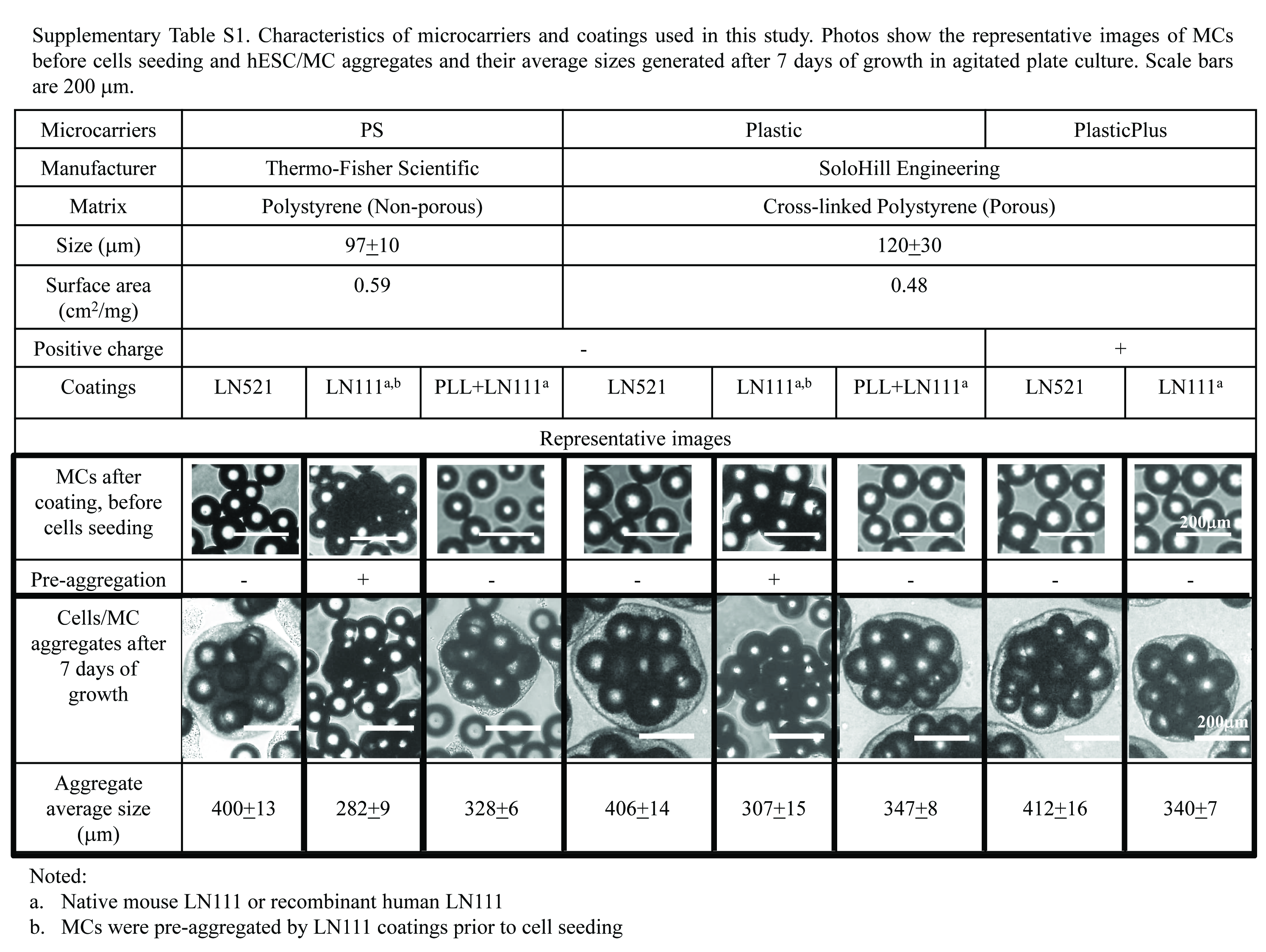

Supplement: Supplemental data [file Supp_Table1.tif]

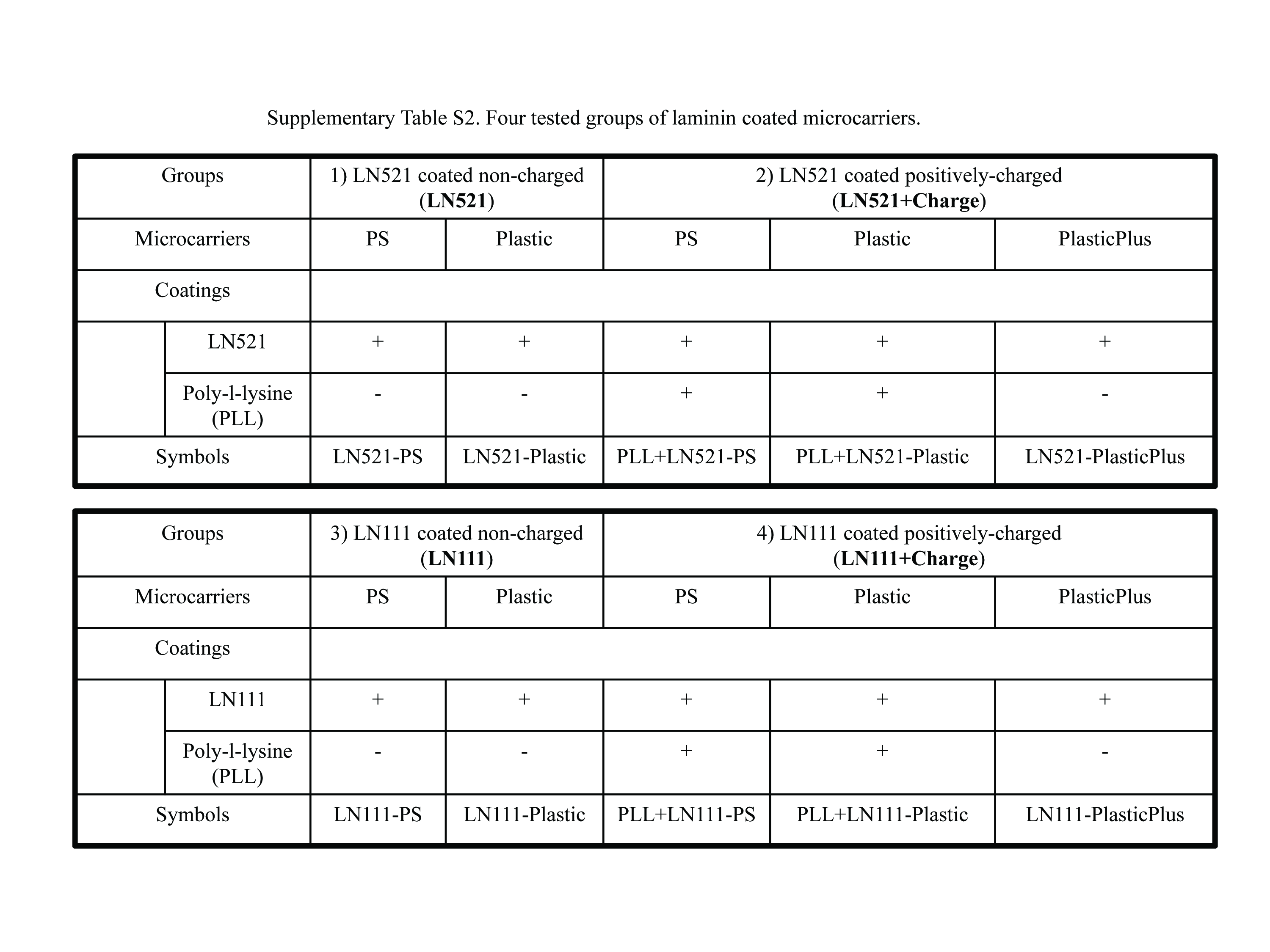

Supplement: Supplemental data [file Supp_Table2.tif]

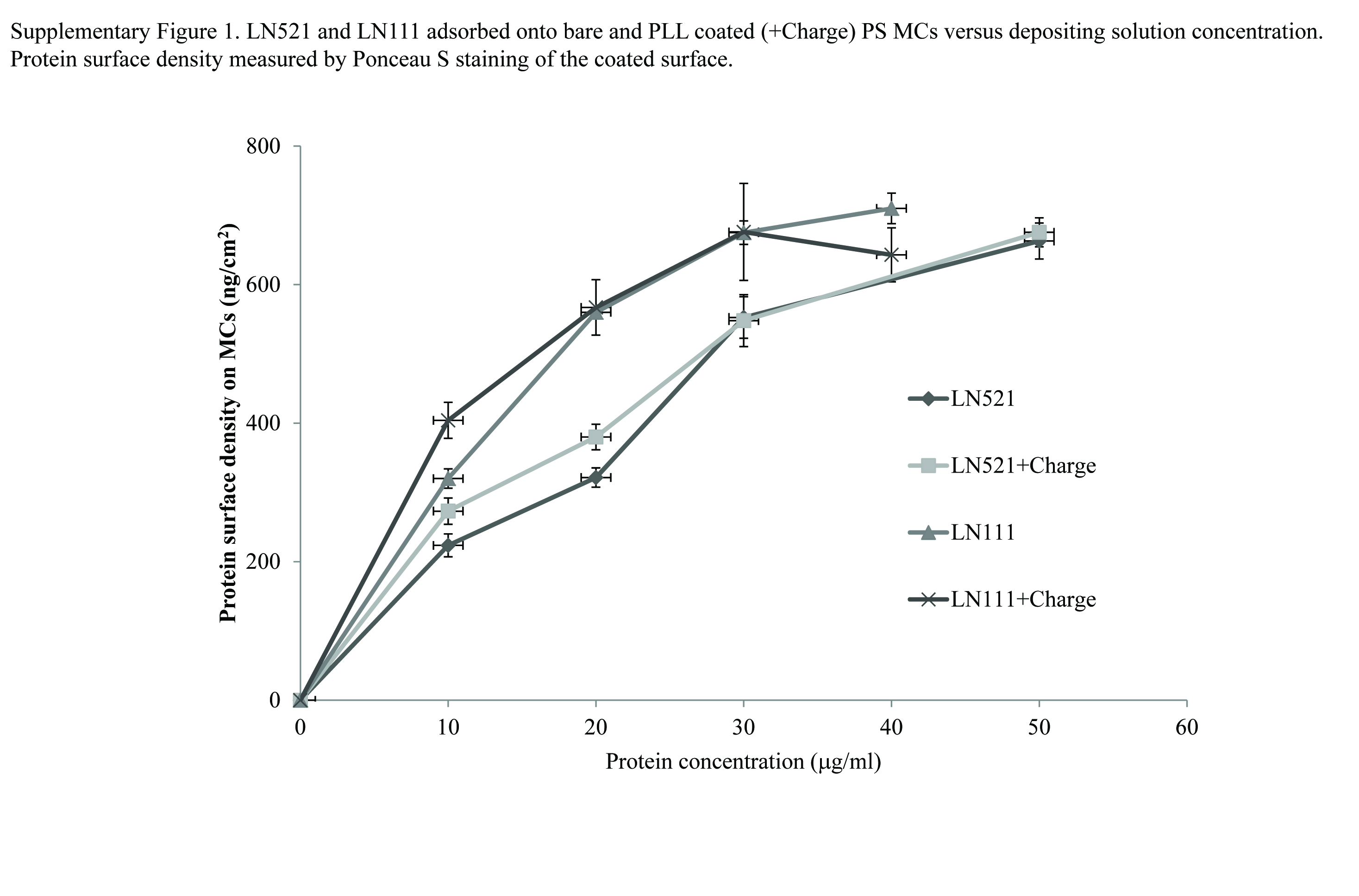

Supplement: Supplemental data [file Supp_Fig1.tif]

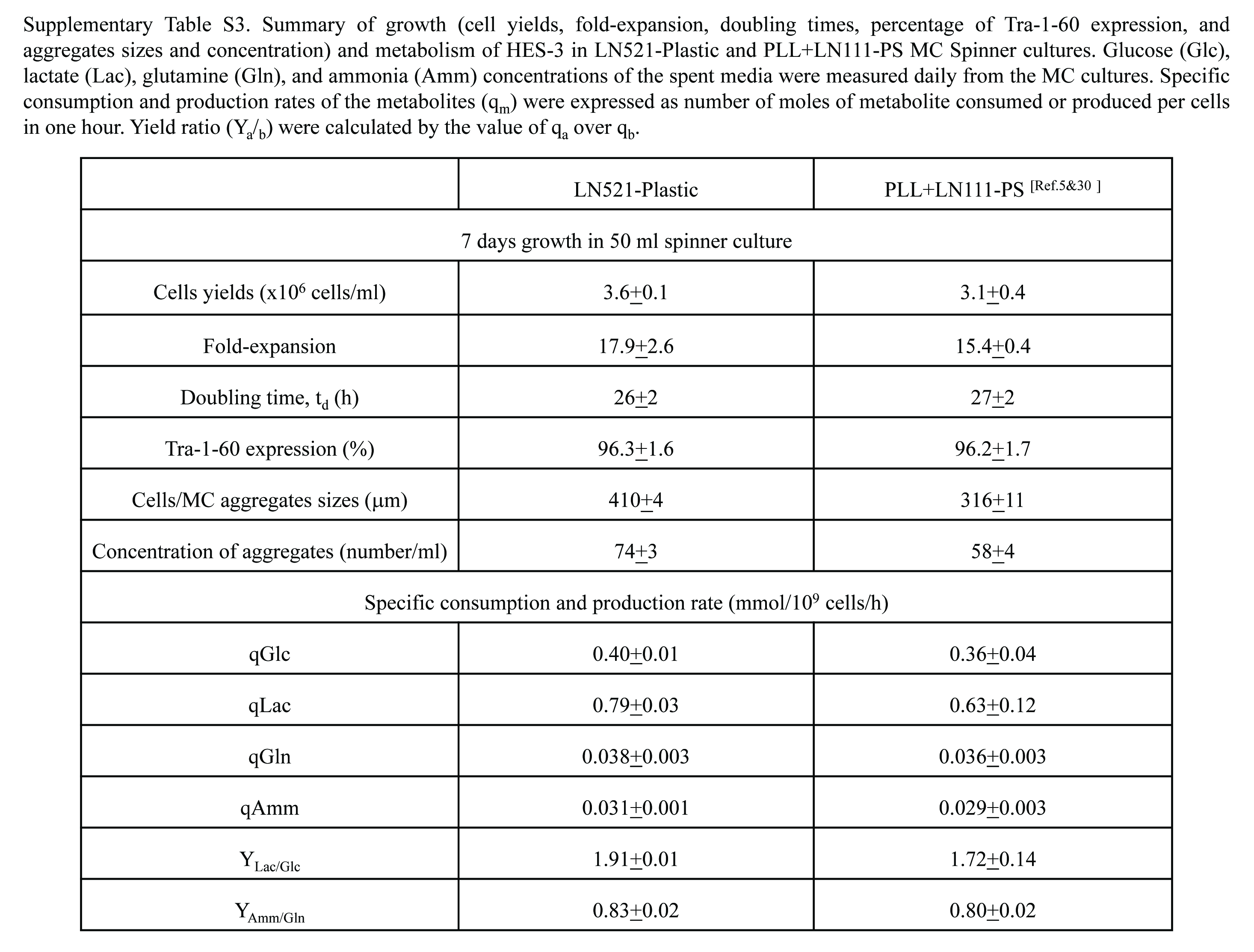

Supplement: Supplemental data [file Supp_Table3.tif]

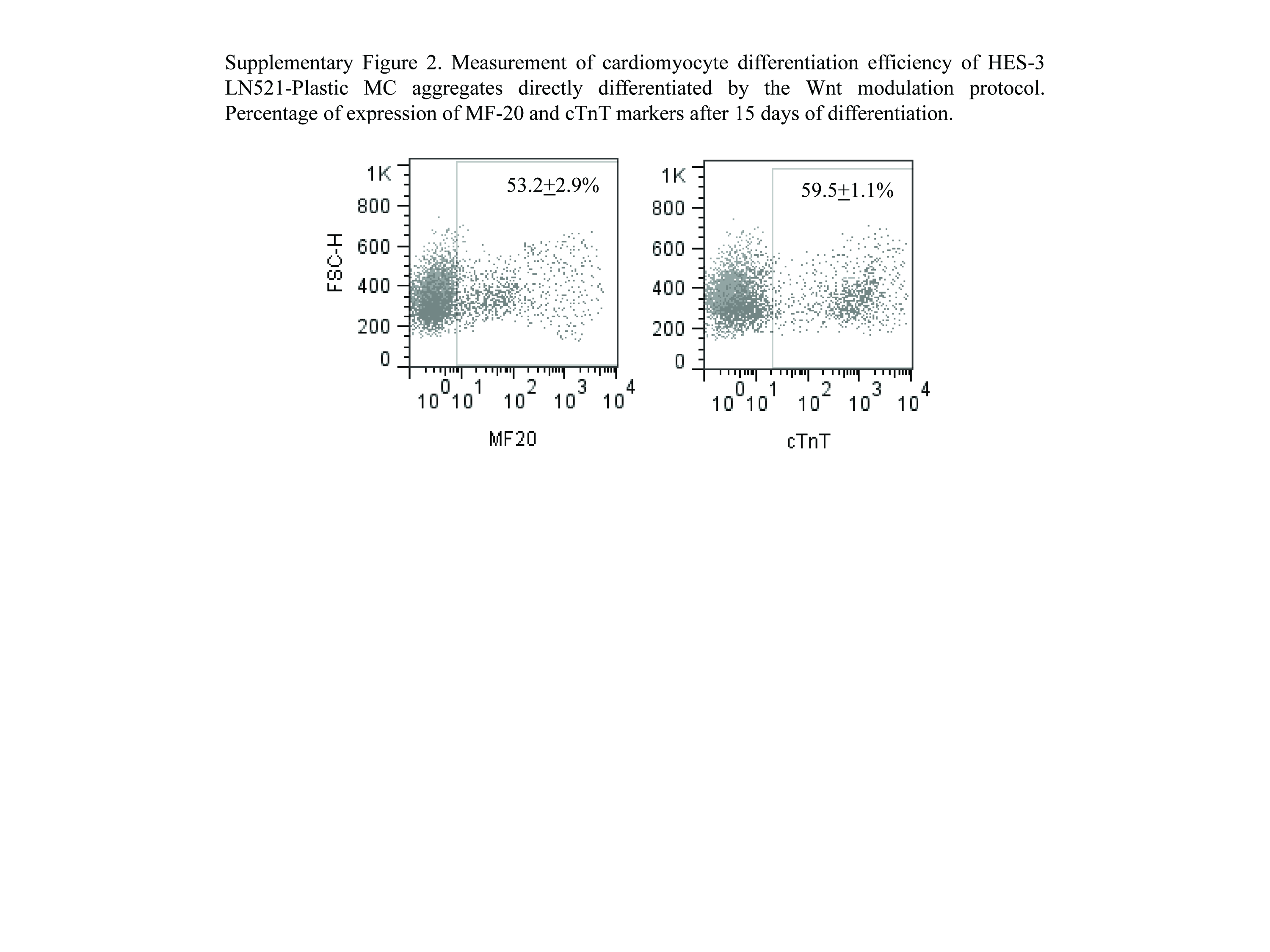

Supplement: Supplemental data [file Supp_Fig2.tif]

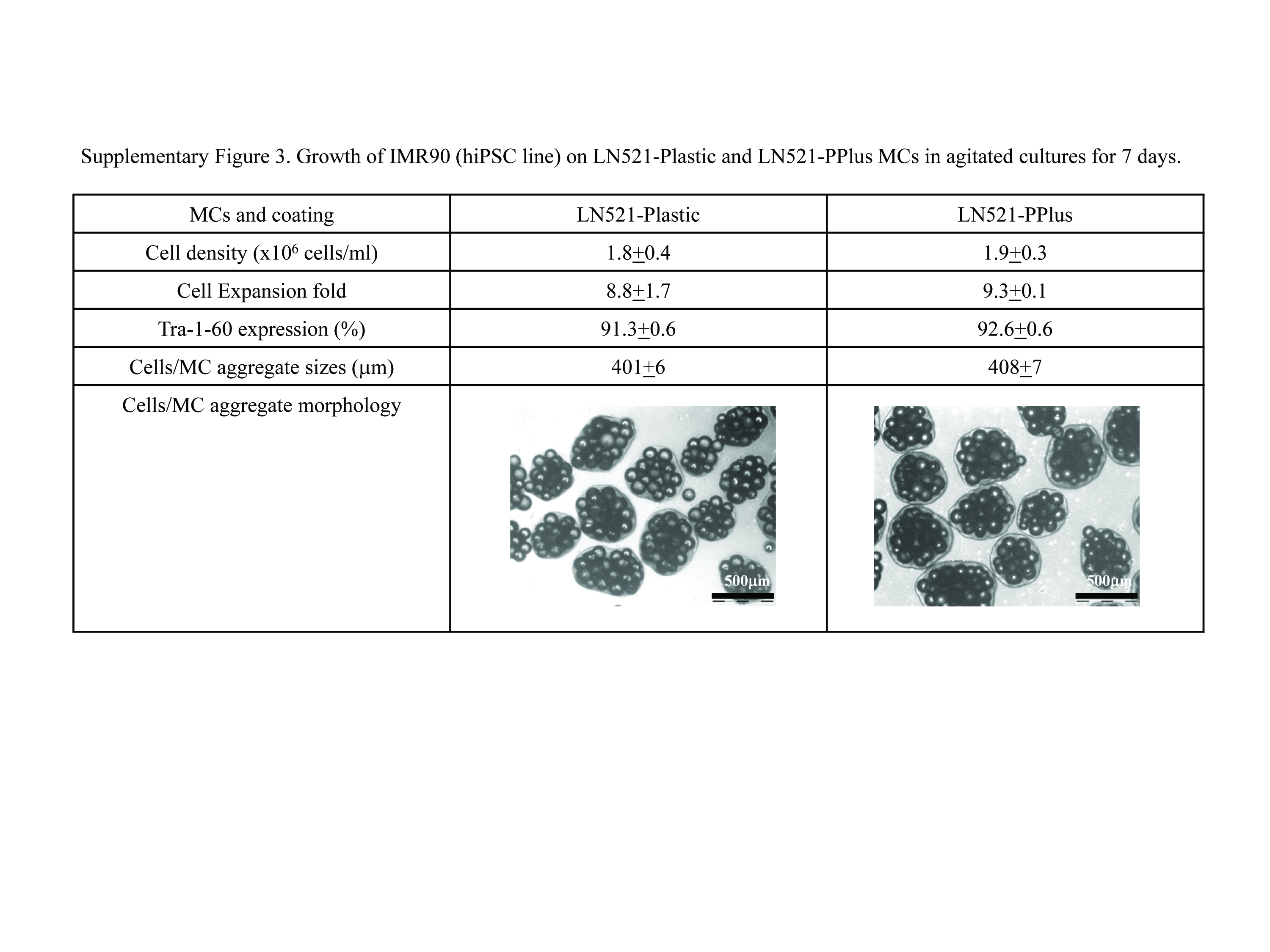

Supplement: Supplemental data [file Supp_Fig3.tif]

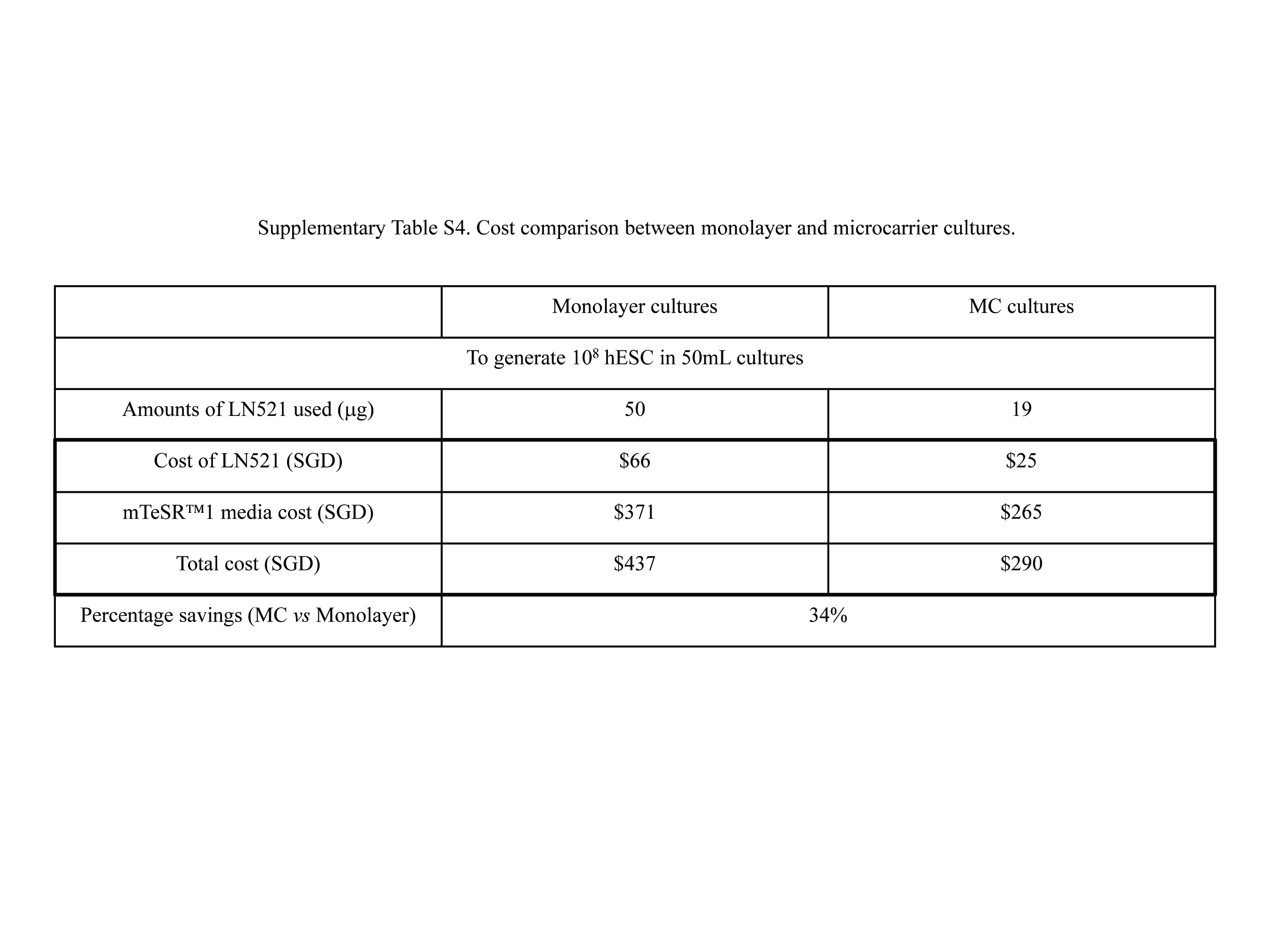

Supplement: Supplemental data [file Supp_Table4.tif]
